# Preexisting psychological illness and its association with mortality in lung cancer patients with access to support resources

**DOI:** 10.1111/crj.13547

**Published:** 2022-10-04

**Authors:** Allison E. Wright, Elyce Sheehan, Fares Qeadan, Lily Stalter, Ali Imran Saeed

**Affiliations:** ^1^ Department of Internal Medicine University of New Mexico Albuquerque New Mexico USA; ^2^ Department of Internal Medicine, Division of Pulmonary Critical Care and Sleep Medicine University of New Mexico Albuquerque New Mexico USA; ^3^ Parkinson School of Health Sciences and Public Health Loyola University Chicago Maywood Illinois USA; ^4^ Department of Surgery University of Wisconsin School of Medicine and Public Health Madison Wisconsin USA; ^5^ Interventional Pulmonary and Advanced Diagnostics Dignity Health Norton Thoracic Institute Phoenix Arizona USA

**Keywords:** disparity, lung cancer, mortality, New Mexico

## Abstract

**Introduction:**

Diagnosis of lung cancer often results in tremendous stress for most patients, especially in patients with underlying psychological illness. Psychosocial support (consultation with psychologist, psychotherapist, or social worker) referral is considered standard for quality cancer care; however, which patients utilize these resources and how these resources affect patient outcomes remain unclear.

**Objectives:**

We aimed to identify which newly diagnosed lung cancer patients accessed available psychosocial resources and assessed how utilization of these resources correlated with treatment and survival outcomes.

**Methods:**

Data were collected from National Cancer Institute‐designated cancer center at the University of New Mexico. We analyzed lung cancer registry and mortality data at the cancer center and bronchoscopy suite data to retrospectively identify patients diagnosed with lung cancer between 2012 and 2017. We used a logistic regression model to compare psychological support utilization at the cancer center between patients with and without history of psychiatric illness. We used a Cox proportional hazards model to identify individual risk factors for mortality.

**Results:**

Patients with a previous psychological diagnosis were 2.4 times more likely (odds ratio = 2.443; confidence interval [CI], 1.130–5.284) to utilize psychological resources than patients without a pre‐cancer psychological diagnosis. Patients who received psychosocial intervention had a 120.4% higher hazard of dying than those who did not (hazard ratio = 2.204; 95% CI, 1.240–3.917). One‐year survival probability among those who did not utilize resources was 62.65% (95% CI, 55.24%–71.06%) and 43.0% (95% CI, 31.61%–58.50%) among those who did. Patients with a previous psychiatric diagnosis were more likely to utilize psychosocial resources within 1 year of lung cancer diagnosis.

**Conclusions:**

Patients with previous psychiatric illness are more likely to utilize psychosocial resources at the cancer center after a new diagnosis of lung cancer. Patients who utilize psychosocial interventions have higher 1‐year mortality than those who do not.

## Graphical abstract

Diagnosis of lung cancer often results in tremendous stress for most patients, especially in patients with underlying psychological illness. Psychosocial support (consultation with psychologist, psychotherapist or social worker) referral is considered standard for quality cancer care; however, which patients utilize these resources and how these resources affect patient outcomes remains unclear.

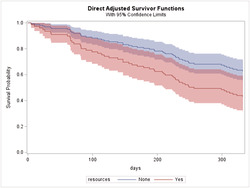

## 1 BACKGROUND

A cancer diagnosis, regardless of type, has been shown to cause a significant amount of stress in a patient. Such stress may manifest as anxiety, depression, exacerbation of other psychiatric symptoms or a combination thereof. Reports of depression and anxiety symptoms in patients diagnosed with cancer range from 12% to 43%.^1^ In patients diagnosed with lung cancer specifically, depression and anxiety symptoms have been described in as many as 28% to 44% of patients.^2^, ^3^ Furthermore, a study by Kisely et al. showed a higher incidence of lung cancer in patients who had a history of psychiatric illness.^4^ Therefore, it is likely that patients with lung cancer are more likely to develop symptoms of psychiatric illness or to have a preexisting psychiatric illness at the time of cancer diagnosis.

The association between psychiatric symptoms and cancer‐related mortality varies. Multiple studies have shown an increased mortality rate from lung cancer in patients with pre‐cancer depression symptoms or new‐onset depression symptoms compared with those with no depression symptoms.^2^, ^5^ Conversely, another study showed that cancer‐related mortality rates were the same regardless of whether an underlying psychiatric disorder existed.^6^

The data are conflicting regarding the association between psychiatric illness and lung cancer mortality; however, depression, anxiety, and/or underlying psychiatric illness during cancer treatment have consistently been associated with reduced adherence to treatment plans, diminished quality of life, longer hospital stays, higher number of re‐admissions, and greater enrollment in hospice programs.^2^, ^7^, ^8^ Considering the profound association between psychiatric symptoms and cancer care and the increased frequency of psychiatric symptoms among lung cancer patients, resources to address underlying psychiatric symptoms are of paramount importance in the treatment of lung cancer. Less than half of cancer patients who experience stress are actually identified and referred for the appropriate treatment.^3^, ^9^

This study aimed to identify newly diagnosed lung cancer patients who accessed psychosocial resources (i.e., social work, psychology, or psychiatry) and to determine whether utilization of such resources correlated with survival probability within the first year of diagnosis at a National Cancer Institute (NCI)‐designated cancer center.

## 2 METHODS

This study was conducted in accordance with the amended Declaration of Helsinki. Data collection and study protocol were approved by and performed in compliance with the Institutional Review Board at the University of New Mexico and University of New Mexico Cancer Center.

### 2.1 Patient selection

Patients were retrospectively identified using data from University of New Mexico. Collected data included bronchoscopy suite data and lung cancer registry of patients who underwent treatment at the cancer center. We included patients diagnosed with lung cancer between 2012 and 2017. Patients' social and medical histories were evaluated by review of electronic medical record. Patients were considered to have a preexisting psychiatric illness (anxiety, depression, schizophrenia, and/or bipolar disorder) if a psychiatric illness was listed in their past medical history, or if they had previously been prescribed anxiolytics, antidepressants, or antipsychotics.

### 2.2 Resource utilization

Utilization of psychological resources was evaluated based on presence of progress notes from social work, psychology, or psychiatry departments during the time of cancer treatment up to 1 year after cancer diagnosis in the electronic medical record. A logistic regression model was used to compare psychological resource utilization between those with and without previous history of psychiatric illness.

### 2.3 One‐year mortality evaluation

Using Cox proportional hazards modeling, the following predictors were used to evaluate the hazard of dying from lung cancer: utilization of resources (yes vs. no), type of lung cancer (neuroendocrine vs. adenocarcinoma/squamous), chemotherapy status (received vs. not received), radiation status (received vs. not received), insurance type (Medicare, Medicaid, [institution blinded for review] Care, private, or Veterans Affairs [VA] insurance), race (White vs. non‐White), sex (male vs. female), age (65 to 85 years vs. 86 years or older), and stage of cancer (≤IIB vs. ≥IIIA). We note that patients who received surgery were included in the study.

## 3 RESULTS

We identified 188 patients who were diagnosed with lung cancer at the bronchoscopy suite at the University of New Mexico and subsequently received treatment at the University of New Mexico Cancer Center over a 6‐year time period. We confirmed the diagnosis using the cancer center lung cancer registry. Date of death was identified using the electronic medical record and condolence registry at the cancer center. Only 186 of the 188 patients had complete data. Patient characteristics are summarized in Table 1.

**TABLE 1:** Demographics of 188 patients diagnosed with lung cancer

Variable	*n* (%)
Gender	
Male	115 (61.2)
Female	73 (38.8)
Age, years	
≤65	84 (44.7)
66–85	98 (52.1)
≥86	6 (3.2)
Race	
White	136 (72.3)
Hispanic	29 (15.4)
Native American	4 (2.1)
Other	16 (8.5)
Unknown	3 (1.6)
Type of cancer^a^	
Adenocarcinoma	94 (51)
Squamous	62 (33)
Neuroendocrine	30 (16)

^a^ Data reflect type of cancer in 186 patients, as patient data were incomplete for 2 patients.

### 3.1 Resource utilization

Sixty‐six of the 186 patients (35.5%) had a pre‐cancer psychiatric diagnosis, whereas 120 patients (64.5%) had no such previous psychological diagnosis. After adjusting for age and type of lung cancer, patients with a previous psychological diagnosis were found to be 2.4 times more likely (odds ratio = 2.443; confidence interval [CI], 1.130–5.284) to utilize stress management resources compared with patients without a pre‐cancer psychological diagnosis (Table 2).

**TABLE 2:** Resource utilization regressed against previous psychological diagnosis, adjusted for age and type of cancer

Independent variable	Odds ratio	95% confidence interval
Psychological history		
Previous psychological diagnosis vs. no previous diagnosis	2.443	(1.130–5.284)
Age		
≤65 vs. ≥86	1.005	(0.104–9.680)
66–85 vs. ≥86	2.266	(0.246–20.897)
Type of cancer		
Adenocarcinoma/squamous vs. neuroendocrine lung cancer	2.026	(0.726–5.7657)

### 3.2 Mortality

**FIGURE 1:**
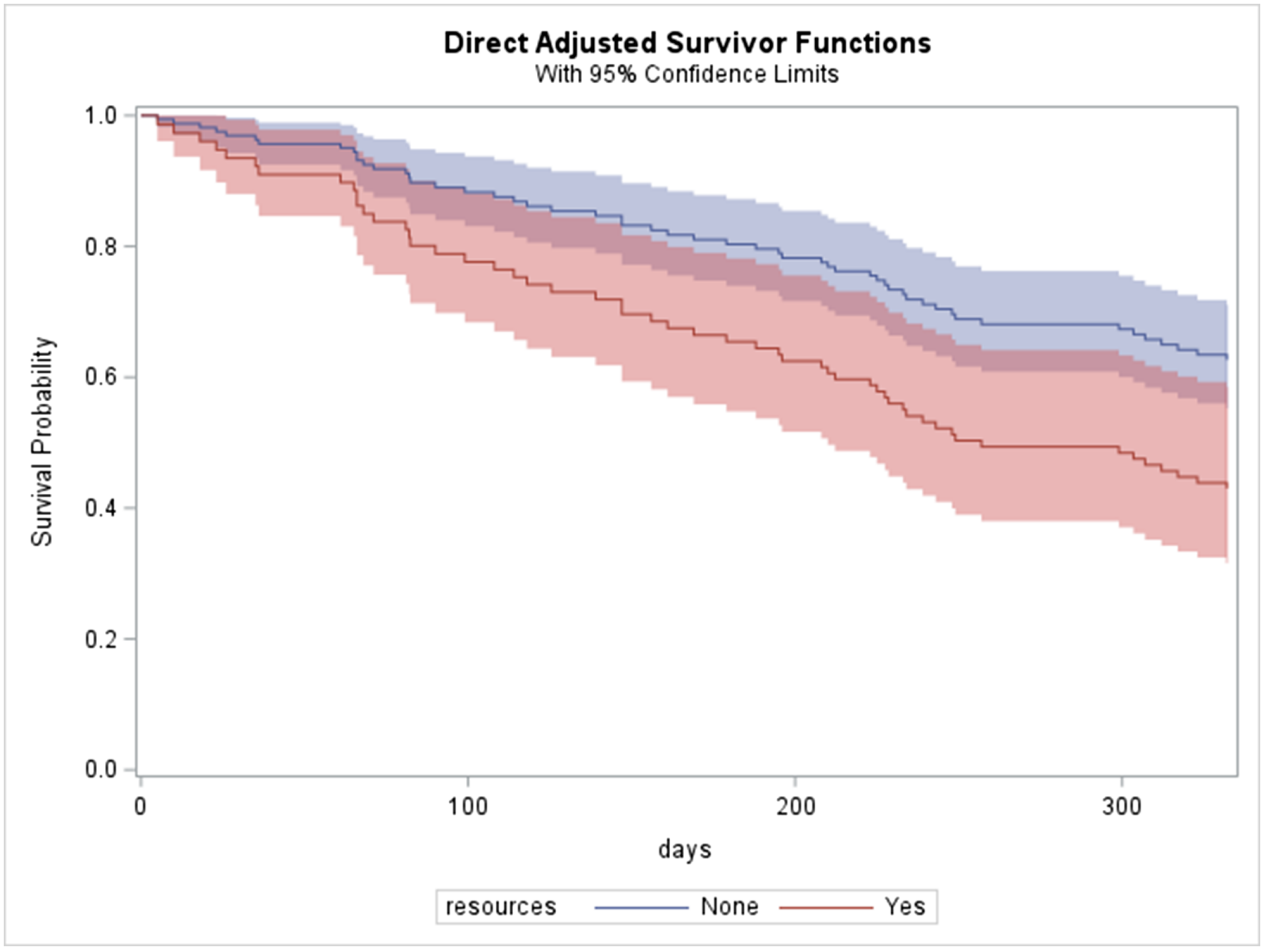
Survival probability within a year of cancer diagnosis by utilization of resources while adjusting for cancer stage, chemotherapy, and race. Statistical difference is observed based on the log‐rank test (*χ* ^2^ = 7.2596, *p* = 0.0071).

Of the 186 patients diagnosed with lung cancer, 68 patients (36.6%) died within 12 months of diagnosis. When we analyzed utilization of resources, type of lung cancer (neuroendocrine vs. adenocarcinoma/squamous), chemotherapy status, radiation status, insurance type, race, sex, age, previous psychiatric diagnosis, and cancer stage, we found that patients who saw a social worker, a psychologist, or a psychiatrist had a roughly 120.4% higher hazard of dying than those who did not (hazard ratio = 2.204; 95% CI, 1.240–3.917). The 11‐month survival probability of patients who did not utilize psychological support resources was 62.65% (95% CI, 55.24%–71.06%) but was only 43.0% (95% CI, 31.61%–58.50%) for those who did (Figure 1). In a sub‐group analysis, we identified cancer stage, chemotherapy status, and race as independent risk factors for mortality.

## 4 DISCUSSION

A strong association has been demonstrated between lung cancer and symptoms of psychiatric illness. Psychiatric illness is an independent risk factor for lung cancer, and new diagnosis of lung cancer itself is known to cause a significant amount of stress in the form of depression, anxiety, and/or exacerbation of underlying psychiatric illness.^1^, ^2^, ^3^, ^4^ Screening patients for stress and offering psychosocial intervention are considered standard of care for patients newly diagnosed with lung cancer. We sought to determine whether certain newly diagnosed lung cancer patients were more likely to access psychosocial resources and to understand how the utilization of those resources correlated with survival probability within the first year of diagnosis.

The University of New Mexico Cancer Center is one of 49 centers in the United States with a comprehensive designation from the NCI. Every new cancer patient is provided with a dedicated nurse and a nurse navigator. There is also an on‐site psychologist for immediate consultation.

### 4.1 Utilization of resources

In the current study, we found that preexisting psychiatric illness affected 36% of the patients who were newly diagnosed with lung cancer. We also found that patients with a preexisting psychiatric diagnosis were 2.4 times more likely to utilize psychosocial resources compared with those without a preexisting diagnosis of psychiatric illness seeking cancer care at our NCI‐designated cancer center. We hypothesize that there may be a number of different causes for the difference in resource utilization between patients with and without a previous diagnosis of psychiatric illness.

First, the social stigma associated with psychiatric illness may prevent patients with new diagnosis of lung cancer from discussing their symptoms. This may be especially true for patients without a preexisting psychiatric diagnosis. Additionally, patients may not recognize when their symptoms of distress meet the criteria for a diagnosis of depression, anxiety, or another type of psychiatric illness. A study by Mosher et al. found that 57% of lung cancer patients with anxiety or depression (based on the Hospital Anxiety and Depression Scale) did not access mental health services, as they did not recognize the need for help or felt that they did not need help.^9^

Another factor that may explain the difference in resource utilization between patients with and without a previous diagnosis of psychiatric illness is awareness of available resources. Previous studies have cited lack of awareness as a barrier to psychosocial interventions in approximately 20% of cancer patients.^9^, ^10^ Lack of awareness would likely be more prevalent in patients who had no previous psychiatric diagnosis, as they likely have not previously used mental health resources compared with patients with preexisting psychiatric illness.

Another aspect to consider is that modern screening methods in clinics may not recognize stress symptoms. Although it is becoming standard of care for cancer patients to be screened for psychiatric illness symptoms during cancer treatment, a study found that only 49% of patients recall being asked about mental stress, worry, or mood changes at their follow‐up oncology appointments.^11^ A study by Mitchell et al., focusing more on clinical practices, found that only 63% of clinicians regularly or always screened for distress or depression. That same study further assessed the type of stress screening being performed, and the researchers found that 76% of practitioners asked only two to three questions or used an ultra‐short survey to screen for symptoms of anxiety or depression.^12^ We speculate that patients with a known psychiatric history are screened more closely for exacerbation of their psychiatric illness, resulting in greater recognition of symptoms. Another commonly cited reason for not utilizing psychological support resources is lack of physician referral.^9^, ^10^ Physicians may have a lower threshold to refer those with a history of psychiatric illness than those without. Furthermore, race has been shown to play a role in physician referral; 32.9% of non‐White patients report a lack of physician referral as a barrier to psychosocial treatment, but only 20.4% of White patients report the same.^10^ This may play a larger role in a state like New Mexico, where a large percentage of the population identifies as non‐White.^13^

Insurance and cost of psychological resources have been offered as possible barriers to referral for stress support; however, studies have found that only 5% to 8% of patients identify cost as a barrier to psychosocial treatment.^10^ In New Mexico, however, we suspect that cost would be cited more frequently, given New Mexico's below‐average socioeconomic status.^14^

### 4.2 Mortality

Studies that have examined this relationship have reported varying results. Arrieta et al. found that, in patients with late‐stage, non‐small‐cell lung cancer (Stages IIIB/IV), depression was an independent risk factor for mortality whereas anxiety was not.^1^ Vodermaier et al. reported that both anxiety and depression were independent risk factors for mortality in those with Stage III non‐small‐cell lung cancer.^15^ Another study focusing on the progression of psychiatric symptoms found increased cancer‐related mortality in patients diagnosed with depression and early stage lung cancer (I/II); however, those researchers found no difference in mortality in patients with late‐stage cancer.^2^ Schizophrenia in patients with lung cancer has been associated with a higher mortality rate in multiple studies.^16^, ^17^ These conflicting results are likely related to variations in study design, making the studies difficult to compare directly.

Regardless of these contradictory results, multiple studies have consistently demonstrated that stress symptoms in cancer patients lead to longer hospital stays, more re‐admissions, and reduced quality of life.^7^ As a result, psychosocial interventions have become imperative in the treatment of patients with lung cancer; however, very little research has investigated how psychosocial intervention affects mortality. Our study found that patients who accessed psychosocial resources, regardless of psychiatric history, had a significant increase in mortality within 1 year of their lung cancer diagnosis. These results contradict the only other study that also examined this relationship. That study, conducted by Duggan et al., found that psychosocial intervention had no significant impact on survival in lung cancer patients. However, this study was performed outside the United States, in South Western Sydney, and was limited to patients with Stage IV non‐small‐cell lung cancer; thus, comparisons across these studies are limited.^18^ Possible hypotheses to explain increased mortality in our study include increased hospice or palliative referrals from psychosocial providers, resulting in less aggressive care; or psychosocial interventions, resulting in patients opting for less aggressive treatment. Lastly, patients with preexisting psychiatric illness have compliance issues with cancer treatment.

### 4.3 Study limitations

The main limitation in our study is that it is a single‐institution study; therefore, we were restricted to data available in the University of New Mexico electronic medical record system. If patients sought psychosocial care outside the University of New Mexico system and if that was not denoted in the chart, it would not be captured in this report.

The study population is small, which means that subgroup and multivariable analyses might have limited power and therefore limited reliability.

## 5 CONCLUSIONS

Our study suggests that patients with pre‐cancer psychiatric illness are more likely to utilize psychosocial resources, highlighting the need for improved screening for stress and psychological illness. Additionally, we observed decreased survival probability in those who sought psychosocial intervention. It is important to identify patients with higher utilization of psychosocial resources as an independent risk factor for increased mortality and to initiate a patient‐focused cancer treatment plan to help with compliance and minimize psychological emergencies leading to hospitalizations and interruption in lung cancer care. It is important to understand how our interventions are affecting patient well‐being and, ultimately, mortality. Future studies should focus on understanding the mechanisms of emotional distress in a patient with a new lung cancer diagnosis and how psychosocial interventions affect patients' decision‐making, cancer treatment patterns, and, ultimately, mortality.

## CONFLICT OF INTEREST

No conflicts of interest exist for the following authors: Allison E. Wright, Elyce Sheehan, Fares Qeadan, Lily Stalter, and Ali Imran Saeed.

## AUTHOR CONTRIBUTIONS

Ali Saeed, Elyce Sheehan, Allison Wright, Fares Qeadan, and Lily Stalter contributed substantially to the study design, data analysis and interpretation, and the writing of the manuscript.

## ETHICS STATEMENT

Data collection and study protocol were approved by and performed in compliance with the Institutional Review Board at the University of New Mexico and University of New Mexico Cancer Center.

## Data Availability

The data that support the findings of this study are available on request from the corresponding author. The data are not publicly available due to privacy or ethical restrictions.
